# Symbiotic bacteria associated with different species of *Curculio* (Coleoptera: Curculionidae) and their host plants

**DOI:** 10.3389/fmicb.2025.1531847

**Published:** 2025-03-14

**Authors:** Yingshan Liu, Yue Ying, Yan Li, Wei Zhang, Jinping Shu

**Affiliations:** Research Institute of Subtropical Forestry, Chinese Academy of Forestry, Hangzhou, China

**Keywords:** symbiotic bacteria, *Curculio*, host adaptation, host-microbe association, bacteria composition

## Abstract

Bacteria often play important roles in the host adaptation of phytophagous insects. Beetles of the genus *Curculio* (Coleoptera: Curculionidae) include pest species that bore into the seeds of trees in the family Fagaceae and damage the cotyledons. At present, there are few studies of the taxonomic diversity and functional effects of symbiotic bacteria involved in changes in host ranges and host adaptation of *Curculio*. Here, we used 16S rRNA gene Illumina and metagenomic sequencing to compare the composition and functions of the bacterial communities of three species of host plants and several *Curculio* species combinations: *Curculio bimaculatus* feeding on *Castanopsis sclerophylla*, *C. bimaculatus* feeding on *Castanopsis tibetana*, and *Curculio davidi* feeding on *Ca. tibetana*. The host plants influenced the diversity of symbiotic bacteria, while the *Curculio* species influenced the community structure of the symbiotic bacteria. Functional predictions showed that symbiotic bacteria contributed to the metabolism of the hosts. However, consistent with the variation in bacteria, the major metabolism-related bacterial genera varied among the treatment groups. Comparisons of metabolic enzymes based on KEGG (Kyoto Encyclopedia of Genes and Genomes) annotation revealed differences in the enzymes involved in insect development and detoxification of plant secondary compounds among the three groups, and the patterns were influenced by the dominance of the *Curculio* species on the host plants. This study provides valuable insights into the possible role of symbiotic bacteria in *Curculio* as host insects.

## Introduction

The interactions and ecological relationships among plants, phytophagous insects, and symbiotic microorganisms have been widely studied; in particular, the role of bacteria in host adaptation and differentiation of phytophagous insects has attracted recent attention (Biere and Tack, 2013; Douglas, 2015; Luan, 2024). During a host shift or host range expansion, phytophagous insects establish adaptations to the nutritional structure, plant metabolites, microclimate, competing species, and natural enemies in the new environment, and bacteria often play an important role in this process (Wielkopolan and Obrȩpalska-Stȩplowska, 2016; Stencel and Wloch-Salamon, 2018; Monticelli et al., 2019).

Symbiosis refers to all microorganisms residing within the host insect, encompassing both mutualistic and parasitic relationships. Symbiotic bacteria coexist with insects within their bodies and even within their cells. Symbiotic bacteria can improve the host’s adaptation to the plants they feed on by providing essential amino acids, participating in nitrogen metabolism, or metabolizing toxic secondary metabolites of plants. In bed bugs (*Cimex lectularius*), *Wolbachia* supplies the host with vitamin B essential for survival. Antibiotic treatments to eliminate *Wolbachia* reduced the size of *Cimex lectularius* and prevented their reproduction (Hosokawa et al., 2010). Similarly, the primary endosymbiotic bacterium *Wigglesworthia glossinidia* helps tsetse fly (*Glossina* sp.) to synthesize vitamin B that they lack in their blood meal (Attardo et al., 2020). When green peach aphid (*Myzus persicae*) shifts to cultivated tobacco (*Nicotiana tabacum*), the expression of genes related to enzymes detoxifying nicotine is dramatically increased in its symbiotic bacterium *Buchnera aphidicola*, thereby allowing the host to overcome the toxicity of nicotine and successfully adapt to feeding on the host plant (Singh et al., 2020). The degradation of cellulose, pectin, and lignin in plants by phytophagous insects depends on enzymes produced by symbiotic bacteria (Zhou et al., 2017; Salem and Kaltenpoth, 2022; García-Lozano and Salem, 2024). Symbiotic bacteria can also improve the host’s tolerance to high temperatures or resistance to pesticides (Benoit et al., 2015; Ceja-Navarro et al., 2015; Cornwallis et al., 2023). Symbiotic bacteria can modulate the host resistance to insecticides by affecting the expression of detoxifying enzyme genes such as the cytochrome P450s (P450s) and glutathione S-transferase (GST) gene families (Wu et al., 2020; Zhang et al., 2021). Analyses of insecticide resistance genes in the brown planthopper (*Nilaparvata lugens*) showed that differences in the expression levels of these genes were highly correlated with the abundance of their core symbiotic bacterial communities (Zhang et al., 2023). In addition, symbiotic bacteria can also affect various aspects of host reproduction and growth, including egg production, the sex ratio, and offspring survival (Fukatsu et al., 2001; Harumoto et al., 2016; Shi et al., 2021). *Wolbachia* can induce feminization, leading to parthenogenesis, male killing, or cytoplasmic incompatibility (Werren et al., 2008). In summary, symbiotic bacteria affect the reproduction and adaptation of host insects in various ways. In contrast, the symbiotic bacterial community structure of insects is remodeled in the process of adaptation to the host plant. Phytophagous insects can take up symbiotic bacterial species from the host plants or the environment (Kikuchi et al., 2007; Hannula et al., 2019). Host plants and the environment can shape the diversity and composition of insect symbiotic bacteria (Jones et al., 2019; Ge et al., 2021; Zhang et al., 2024). Such bacteria thus form an important “bridge” in the co-evolutionary relationship between phytophagous insects and their hosts. The study of the diversity and functions of insect symbiotic bacteria is significant for understanding insect-plant interactions.

The genus *Curculio* (Coleoptera: Curculionidae) includes pests of plants in the family Fagaceae. The beetles bore into Fagaceae seeds and damage the cotyledons and thus can seriously affect the regeneration of natural Fagaceae forests (Aoki et al., 2009; Hou et al., 2010; Zhou et al., 2022). More than 200 species of *Curculio* have been reported globally, and they have a wide host range within the Fagaceae. In the co-evolution of *Curculio* and Fagaceae, the host ranges of different species of *Curculio* have clearly differentiated. There are polyphagous species that feed on a wide variety of genera within the Fagaceae, including the weevil *Curculio bimaculatus*, and oligophagous species that can only feed on a particular species or genus of Fagaceae, for example the weevil *Curculio davidi* (Pelsue and Zhang, 2002; Pelsue and Zhang, 2003). Their distinct feeding strategies, which include both oligophagous and polyphagous species, make them an excellent model for studying plant-herbivore interactions and co-evolutionary processes. The variation in the host ranges among species reflects the variation in adaptive ability, but studies on the role of symbiotic bacteria involved in this process are limited. We hypothesized that variation for hosts affected the performance, bacterial community of acorn weevil larvae and the symbiotic bacteria could mediate the ability of host plant adaptation. In this study, we collected polyphagous *C. bimaculatus* feeding on *Castanopsis sclerophylla* and *Castanopsis tibetana* and the oligophagous *C. davidi* feeding on *Ca. tibetana*. We investigated the taxonomic diversity, abundance, and community structure of symbiotic bacteria in different insect and plant combinations. Functional predictions for bacterial communities were made based on metagenomic sequencing. We compared metabolism-related bacterial genera of weevils and their contributions to metabolism pathways. In this study we explored changes in symbiotic bacterial communities in polyphagous or oligophagous weevils, especially when the host plant changes. In addition, which bacteria play a role in this process. This study aims to provide insight into the possible role of symbiotic bacteria in *Curculio* as host insects. These findings contribute to a broader understanding of herbivorous insect adaptation, microbial symbiosis, and plant-insect interactions, which may be of interest to researchers studying insect ecology, evolutionary biology, and microbiome dynamics.

## Materials and methods

### Sample collection and DNA extraction

We collected seeds of *Ca. sclerophylla* and *Ca. tibetana* infested with weevils during October and November 2022 when the leaves were falling and after the weevil larvae had parasitized the seeds. The seeds were kept in plastic boxes in the laboratory where emerging larvae were collected. Healthy *C. bimaculatus* larvae were collected from *Ca. sclerophylla* and *Ca. tibetana*. *Curculio davidi* larvae were collected from *Ca. tibetana*. Individual larvae from different host species were collected and labeled; thus, *C. bimaculatus* feeding on *Ca. sclerophylla* were labeled as BS; *C. bimaculatus* feeding on *Ca. tibetana* were labeled as BT, and *C. davidi* feeding on *Ca. tibetana* were labeled as DT (Table 1). The mature larvae were collected and surface sterilized by washing with ddH_2_O for 3 min to remove surface dust, then soaking in 75% alcohol for 3 min to disinfect the body surface, rinsing with 2% NaClO solution for 30 s, and rinsing three times in ddH_2_O, each time lasting 1 min, to remove residual reagents. After the treatment, each larva was placed in a sterile 2.0 ml centrifuge tube containing two small steel beads (φ = 3 mm) and stored at −80°C. Individual larvae were ground with a tissue grinder (60 Hz, 55–60 s; Tissuelyser-48, Shanghai Jinxin Industrial Development Co., Ltd.) for DNA extraction. Total DNA was extracted from each larva using the TIANamp Genomic DNA Kit (TIANGEN Biotech Co., Ltd.) according to the manufacturer’s instructions. DNA extracts were checked on 1% agarose gels. Since weevil larvae are very similar in appearance, we amplified the DNA fragments of the coding regions of mitochondrial cytochrome oxidase subunits I (COI) to identify each larva to species. The sequences were amplified using the PCR primers LCO1490 (5′-GGTCAACAAATCATAAAGATATTGG-3′) and HCO2198 (5′-TAAACTTCAGGGTGACCAAAAAATCA-3′) (Folmer et al., 1994). Amplification of mtDNA was performed as follows: an initial 1-min denaturation (98°C), followed by 30 cycles of 30 s at 94°C, 30 s at 48°C, and 1 min at 72°C, and a final extension cycle of 5 min at 72°C. All PCRs were performed in a total volume of 25 μL using 2 × Taq PCR MasterMix (TIANGEN Biotech Co., Ltd.), according to the manufacturer’s instructions.

**TABLE 1:** Sample collection sites and host plant species of *Curculio* spp.

Host plant	Longitude	Latitude	Species	Groups
*Castanopsis sclerophylla*	110.3010°	29.4420°	*Curculio bimaculatus*	BS
*Castanopsis tibetana*	109.0406°	29.4445°	*C. bimaculatus*	BT
			*C. davidi*	DT

### 16S rRNA gene amplification and sequencing

The DNA of three larvae with the same labels was combined as one sample. Amplification of the V3-V4 regions of the 16S rRNA gene was performed using the primers 338F (5′-ACTCCTACGGGAGGCAGCA-3′) and 806R (5′-GGACTACHVGGGTWTCTAAT-3′) (Claesson et al., 2010). The PCR comprised an initial denaturation at 95°C for 3 min, followed by 30 cycles of 95°C for 30 s, 55°C for 30 s, 72°C for 45 s, and a final extension step at 72°C for 10 min. All PCRs were performed in a 20 μL total volume using *TransStart*^®^ *FastPfu* DNA Polymerase according to the manufacturer’s instructions. PCRs were performed in triplicate. The PCR products were purified, pooled in equimolar concentrations, and sequenced on the Illumina MiSeq PE300 platform (Illumina, San Diego, CA, USA) at the Majorbio Bio-Pharm Technology Co., Ltd (Shanghai, China). Paired-end sequences were merged into a single sequence with a length of ∼300 bp using FLASH 1.2.11. The obtained sequences were imported into the QIIME2. The DADA2 plugin in was used to denoise sequencing reads, resulting in high resolution amplicon sequence variants (ASVs) for downstream analysis. The consensus sequences for the ASVs were classified with a classify-sklearn classifier trained against the SILVA 16S rRNA gene reference (release 138.2) database.

### Metagenomic sequencing and binning

For each group, DNA extracted from three individuals was selected and pooled to create a composite biological sample, with three such pools (representing three biological replicates) being collected for each group. In total, nine samples were used for metagenomic analyses of the weevils from the three groups. The composite samples were randomized such that the same sample names appeared in the microbiome and metabolomics analyses, but they represented different mixtures of individuals with the same labels. Based on the methods already described (Yuan et al., 2021; Zhou et al., 2023), libraries were prepared by fragmenting the extracted DNA to ∼350 bp using Covaris ME220 (Covaris, USA) and sequencing the paired-end reads on the Illumina HiSeq X-Ten platform at the Majorbio Bio-Pharm Technology (Shanghai, China), and generated a sequencing depth of approximately 12G per sample. Low-quality raw reads, sequences with low-quality bases (length < 50 bp or with a quality value < 20), and adapters were removed using Fastp v0.20.0.^1^ About 12 Gb of raw data were obtained from each sample. MEGAHIT v1.1.2^2^ was used to assemble high-quality reads, and contigs shorter than 300 bp were discarded. After gene prediction with Prodigal v2.6.3,^3^ CD-Hit67^4^ was used to cluster the sequences to create a non-redundant gene catalog with 90% sequence identity and 90% coverage. High-quality reads were aligned to the non-redundant gene catalogs to calculate gene abundance with 95% identity using SOAPaligner^5^ (version 2.21). Taxonomic analyses were performed by comparisons (BLASTp) against the NCBI-NR database at the *e*-value cutoff of 1e^–5^ using Diamond v2.0.13.^6^ Based on the results of taxonomic annotation, a non-redundant gene catalog of bacteria was selected for functional annotation by comparisons (BLASTp) against the KEGG, and COG databases at the *e*-value cutoff of 1e^–5^ using Diamond v2.0.13.

Using assembled metagenomic contigs with a minimum length of 1,000 bp, binning was performed on single-sample data using the CONCOCT tool Version 0.5.0.^7^ All resulting bins were subsequently dereplicated using dRep Version 2.2.9.^8^ The average nucleotide identity (ANI) analysis was performed based on the reference genome using fastANI version 1.0. Primary clusters were established based on a Mash ANI threshold of ≥ 90%. Secondary clustering was then performed at an ANI threshold of ≥ 99%, requiring a genome overlap of ≥ 10% between genomes. Quality evaluation of the resulting metagenome-assembled genomes (MAGs) was conducted using CheckM Version 1.0.12,^9^ following the criteria of completeness ≥ 50% and contamination < 10%. The obtained medium-quality MAGs and genome of one representative symbiotic bacterium *Sodalis* were further analyzed using antiSMASH to predict biosynthetic gene clusters (BGCs).

### Determination of metabolite contents

All seeds without weevil holes of every group were selected from the collected seeds for determination of metabolite contents. Each group of seeds collected were ground into the powder. All content determinations of the seed samples were carried out in triplicate.

The fat content was determined by the Soxhlet extraction method (Latimer and Latimer, 2023). Approximately 5 g of finely ground seed samples were placed in a thimble and subjected to continuous extraction with petroleum ether for 8 h. After extraction, the solvent was evaporated, and the remaining lipid content was weighed to determine the fat percentage.

The protein content was determined by the Kjeldah method (Hagerman and Butler, 1978). 2 g of seed powder was digested in concentrated sulfuric acid with a catalyst, converting organic nitrogen into ammonium sulfate. The digest was then neutralized with sodium hydroxide, and the released ammonia was distilled into a boric acid solution and titrated with hydrochloric acid. The nitrogen content was converted to protein content using a factor of 5.3.

Soluble sugar and starch contents were determined using anthrone-sulfuric acid colorimetry (Latimer and Latimer, 2023). In this assay, 2 g of seed material was hydrolyzed into simple sugars, which reacted with anthrone reagent in the presence of concentrated sulfuric acid, producing a blue-green complex. The absorbance of the reaction mixture was measured spectrophotometrically at 620 nm, and the concentrations of soluble sugar and starch were determined using calibration curves prepared with standard reference (glucose for soluble sugar, starch for starch).

The flavonoid content of the seeds was determined spectrophotometrically using rutin as a standard (Zhishen et al., 1999). A weighed portion of 1 g of seed material was extracted using ethanol, and the total flavonoid content was estimated by its reaction with Aluminum Nitrate Solution and Potassium Acetate Solution, forming a colored complex. The absorbance was recorded at 420 nm, and the flavonoid concentration was calculated based on a standard curve constructed with rutin.

The tannin content of the seeds was determined spectrophotometrically using gallic acid as a standard (Latimer and Latimer, 2023). 2 g of seed powder was extracted using acetone-water, and tannins were quantified based on their reaction with Sodium Tungstate-Sodium Molybdate Mixed Solution and Sodium Carbonate Solution, producing a color change proportional to tannin concentration. The absorbance was measured at 765 nm, and tannin content was determined using a gallic acid standard curve.

## Results

### Differences in metabolite contents

The protein content of *Ca. tibetana* was significantly higher than that of *Ca. sclerophylla*. The contents of soluble sugars, flavonoids, and tannins of *Ca. sclerophylla* were significantly higher than those of *Ca. tibetana*. There were no significant differences in fat or starch contents between the two tree species (Table 2). Data were analyzed by *t*-test using SPSS software (SPSS Inc., USA). Statistical significance was set to *P* < 0.05 (Rakić et al., 2006).

**TABLE 2:** Metabolite contents of seeds of two species in the Fagaceae.

Plants	Protein (g/100 g)	Fat (g/100 g)	Soluble sugar (g/100 g)	Starch (g/100 g)	Flavonoids (mg/g)	Tannins (mg/g)
*Ca. sclerophylla*	2.37 ± 0.06b	0.63 ± 0.12	8.54 ± 0.73a	57.46 ± 2.47	2.43 ± 0.4a	14.24 ± 0.98a
*Ca. tibetana*	3.02 ± 0.1a	0.6 ± 0.17	5.88 ± 0.43b	56.59 ± 2.12	0.68 ± 0.09b	1.86 ± 0.08b

Mean ± SD. Different lowercase letters indicate significant differences (*P* < 0.05).

### Bacteria differed among different host plants and host insect combinations

After subsampling each sample to an equal sequencing depth, 42,883 sequences were obtained per sample. After annotation, 599 ASVs were obtained belonging to 17 phyla, 26 classes, 67 orders, 110 families, and 204 genera.

**FIGURE 1:**
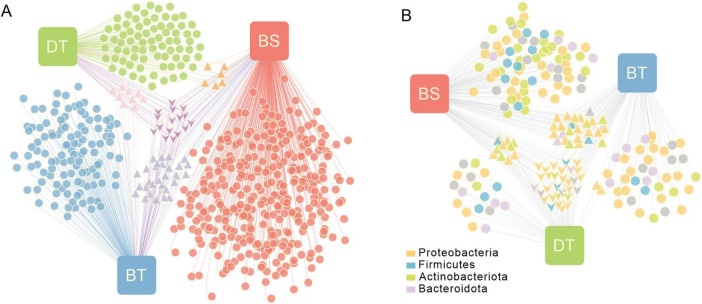
Bipartite association network showing associations between the host plants and the ASVs or bacterial genera. Node sizes represent relative abundance of the ASVs or genera. The edge-weighted spring-embedded algorithm pulled together nodes with similar associations. Triangle-shaped nodes represent nodes associated with two groups. Arrow-shaped nodes represent nodes associated with three groups. BS means *Curculio bimaculatus* feeding on *Castanopsis sclerophylla*; BT means *C. bimaculatus* feeding on *Castanopsis tibetana*; DT means *Curculio davidi* feeding on *Ca. tibetana*, the same as below. **(A)** Associations between the host plants and the 599 ASVs. **(B)** Associations between the host plants and the 204 genera.

An association network was used to visualize the relationships between ASVs and the different host insect or host plant combinations. Of these ASVs, only 2.84% were shared among all groups; 88.6% were associated with only one group. Approximately 1.5% of the ASVs were shared by only two groups from the same host plant *Ca. tibetana*, and 5.01% were shared by two groups of the same host insect *C. bimaculatus*. There were 12.69% and 19.03% unique ASVs detected in the groups from *C. davidi* and *C. bimaculatus*, respectively, feeding on *Ca. tibetana*, and 56.93% unique ASVs were found in *C. bimaculatus* feeding on *Ca. sclerophylla* (Figure 1A). The α-diversity of the samples at the ASV level was estimated by the Chao, Ace, Simpson, and Shannon indices (Supplementary Figure 1A and Supplementary Table 1). The Chao or Ace indices of *C. bimaculatus* feeding on *Ca. sclerophylla* (BS) were higher than the other two groups (Kruskal–Wallis H test, *P* < 0.05). The Simpson indices of *C. davidi* and *C. bimaculatus* feeding on *Ca. tibetana* (DT and BT) were both significantly higher than that of *C. bimaculatus* feeding on *Ca. sclerophylla* (BS; Kruskal–Wallis H test, *P* < 0.001), and the Simpson indices of *C. davidi* feeding on *Ca. tibetana* and *C. bimaculatus* feeding on *Ca. tibetana* were significantly lower than that of *C. bimaculatus* feeding on *Ca. sclerophylla* (Kruskal–Wallis H test, *P* < 0.01). There were no significant differences between the Ace or Chao indices of weevils feeding on the same plants *Ca. tibetana*, but the Shannon and Simpson indices were significantly different between the two groups (Kruskal–Wallis H test, *P* < 0.05). The α-diversity suggested that the bacterial community richness levels and the bacterial community diversity in the weevils feeding on *Ca. sclerophylla* was greater than that in the weevils feeding on *Ca. tibetana*.

The association network of genera showed that the Proteobacteria, Firmicutes, and Actinobacteriota were the three most abundant phyla in the bacterial communities in weevils feeding on Fagaceae seeds (Figure 1B). Of these genera, 32 were shared between all groups; three genera were only shared by groups from the same host plant *Ca. tibetana*; 29 genera were only shared by groups from the same host insect *C. bimaculatus*. The *C. bimaculatus* feeding on *Ca. sclerophylla* had the largest number of unique genera of 74. There were 11 main genera with a total abundance > 1% in nine samples (Supplementary Figure 1B and Supplementary Table 2). The prevalence of *Sodalis*, *Candidatus* Curculioniphilus, *Methylorubrum*, *Rickettsia*, *Paenibacillus* and *Sphingomonas* was 100% (Supplementary Table 2). However, the genera were distributed unevenly among the different groups. *Candidatus* Curculioniphilus, the primary endosymbiont of *Curculio*, had an average abundance > 2.6% in all groups. The *Ca. sclerophylla* feeders showed a greater intrasample bacterial diversity of genera than the *Ca. tibetana* feeders. However, the bacterial composition of *C. bimaculatus* differed from that of *C. davidi*. The β-diversity of samples at the genus level showed that two groups of *C. bimaculatus* were clustered in composition and community structure, while *C. davidi* was an independent cluster (Supplementary Figure 1C). The main genera of *C. davidi* were different from those of *C. bimaculatus*.

### Analysis of the association between bacterial genera and metabolite contents

**FIGURE 2:**
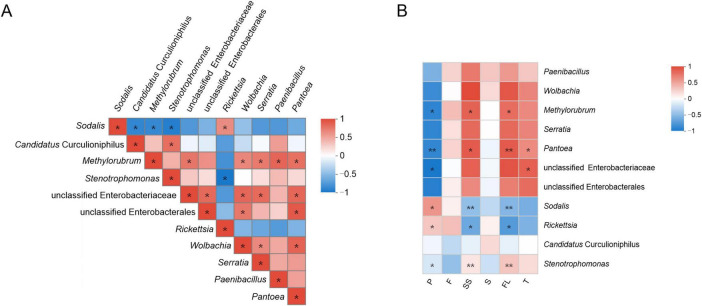
Heatmap showing correlations between dominant bacterial genera or metabolites contents. Positive and negative correlations are indicated as red (0 to 1) and blue (–1 to 0) gradients. **(A)** Spearman correlation coefficients of 11 dominant bacterial genera. **(B)** Spearman correlation coefficients of the top 11 genera and metabolites contents. P means protein; F means fat; SS means soluble sugar; S means starch; FL means flavonoids; T means tannins. The value of *P* < 0.05 is marked with “*”; the value of *P* < 0.01 is marked with “**”.

Spearman’s correlations between bacterial genera with a total abundance > 1% are shown in Figure 2A. The prevalence of *Sodalis*, *Candidatus* Curculioniphilus, *Methylorubrum*, *Rickettsia*, and *Paenibacillus* was 100%. The abundance of *Sodalis* was significantly positively correlated with *Rickettsia*. *Curculio davidi* had higher abundances of *Sodalis* and *Rickettsia* than *C. bimaculatus* (Supplementary Figure 2). The abundance of *Candidatus* Curculioniphilus was significantly positively correlated with *Stenotrophomonas*. The abundance of *Methylorubrum* was significantly positively correlated with *Pantoea*, *Wolbachia*, *Serratia*, *Paenibacillus*, and unclassified Enterobacteriaceae. The *Ca. sclerophylla* feeders showed a high abundance of *Methylorubrum*, *Pantoea, Wolbachia*, and *Serratia*. The abundance of *Pantoea* was significantly negatively correlated with most of the main genera. The *Ca. sclerophylla* feeders had the largest number of genera.

A correlation analysis between the main genera and metabolite contents was visualized using a heatmap (Spearman’s correlations; Figure 2B). Among the four genera with 100% prevalence, the abundances of *Paenibacillus* and *Candidatus* Curculioniphilus had no significant association with the metabolite contents. The abundances of *Methylorubrum* and *Pantoea* in *Ca. sclerophylla* feeders showed a significant negative association with the protein content, and *Methylorubrum* was positively associated with soluble sugars and flavonoids (*P* < 0.05). *Pantoea* was positively associated with tannins (*P* < 0.05). The abundance of *Rickettsia* in *C. davidi* was significantly negatively associated with soluble sugars and flavonoids (*P* < 0.05). There were no genera significantly associated with fat or starch contents.

### Functional predictions for bacterial communities of *Curculio* feeding on Fagaceae seeds

A total of 6,210,941 non-redundant genes were identified in the three groups of samples. After searching for non-redundant gene sequences against the NR database, 99,898 genes were annotated for bacteria. Bacteria comprised the largest percentage of all samples (Supplementary Figure 3A). We selected genes of bacteria based on the results of taxonomic annotation for functional annotation.

**FIGURE 3:**
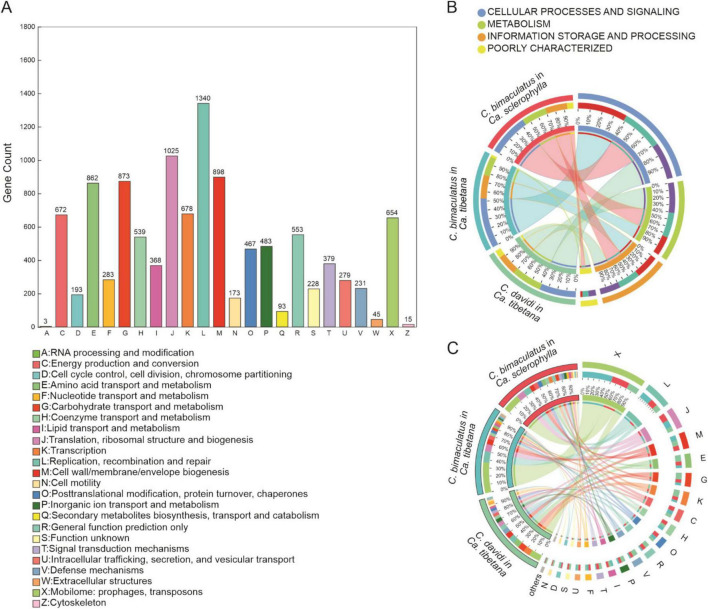
COG analysis for three *Curculio* spp. strains feeding on Fagaceae seeds **(A)** Number of genes annotated to different COG functions. **(B)** Distribution of COG categories for different groups. **(C)** Distribution of COG functions for different groups.

In the COG functional annotations of bacteria, 2,210 COGs belonging to 24 functions were predicted for all samples (Supplementary Table 3). The function with the largest number of COG annotations was [L]Replication, recombination, and repair, followed by [J]Translation, ribosomal structure and biogenesis, [M]Cell wall/membrane/envelope biogenesis, [G]Carbohydrate transport and metabolism, and [E]Amino acid transport and metabolism (Figure 3A). The category with the highest abundance in the COG annotations was cellular processes and signaling, followed by metabolism (Figure 3B). The most abundant function, [X]Mobilome: prophages, transposons, was the same for each group.

In the *C. bimaculatus* groups, the next highest abundances were [L]Replication, recombination, and repair and [J]Translation, ribosomal structure, and biogenesis. However, in the *C. davidi* group, the next highest abundances were [G]Carbohydrate transport and metabolism and [E]Amino acid transport and metabolism, suggesting that the metabolic activity of bacteria in *C. davidi* was stronger (Figure 3C).

**FIGURE 4:**
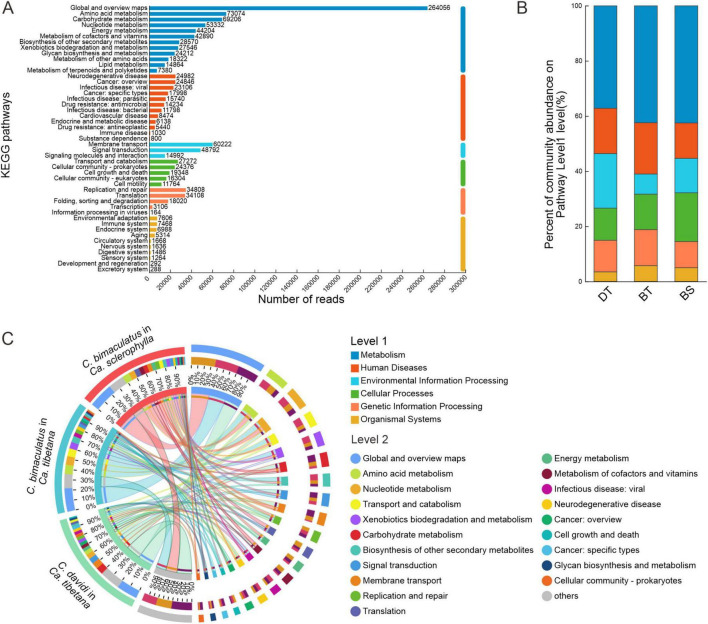
KEGG analysis for three *Curculio* spp. strains feeding on Fagaceae seeds **(A)** Number of reads annotated to different KEGG pathways in level 2. **(B)** Distribution of KEGG pathways in level 1 for different groups. **(C)** Distribution of KEGG pathways in level 2 for different groups.

For the KEGG functional annotations of bacteria, 2,744 KOs (KEGG Orthology) were predicted for all samples (Supplementary Table 4). The KEGG pathway with the largest number of reads was global and overview maps, followed by amino acid metabolism and carbohydrate metabolism (Figure 4A). The proportions of KEGG level 1 pathways were similar across the groups. The most abundant level 1 pathway of each group was the metabolism pathway (Figure 4B). Figure 4C shows the composition of 15 main level 2 pathways in each group. The most abundant level 2 pathway of each group was global and overview maps. The *C. bimaculatus* groups had higher abundances of the amino acid metabolism and nucleotide metabolism pathways. However, *C. davidi* had a higher abundance of the pathways of carbohydrate metabolism and membrane transport. The similar annotation results of COG and KEGG suggest that the *C. davidi* bacterial community has stronger carbohydrate metabolic activity. Overall, the composition of functional genes of *C. bimaculatus* feeding on *Ca. sclerophylla* and *C. davidi* feeding on *Ca. tibetana* was relatively similar in structure (Supplementary Figure 3B).

### Metabolism-related bacterial genera of weevils and their contributions

**FIGURE 5:**
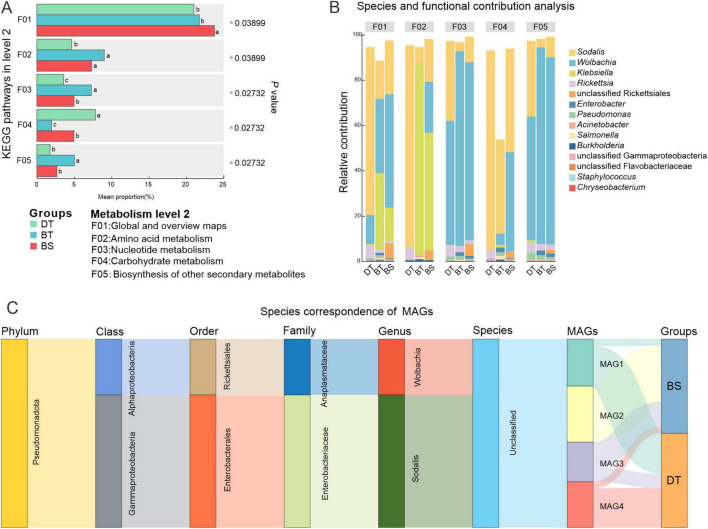
Comparison in metabolism of different groups and contributing bacteria. **(A)** Differences in KEGG pathways of metabolism (Kruskal–Wallis test; *P < 0.05; differences between the two groups are indicated by different letters). **(B)** The relative abundance of a taxon participating in a KEGG function. **(C)** Species correspondence of metagenomic binning MAGs.

The abundance of KEGG level 2 pathways of metabolism was compared between the species to investigate the relative abundances of bacterial genera involved in metabolism-related symbiotic functions in the weevils. There were significant between-group differences in the abundances of five pathways (Figure 5A). The bacteria of *Ca. sclerophylla* feeders harbored a significantly higher abundance of global and overview map genes (Kruskal–Wallis H test, *P* < 0.05). The bacteria of *C. bimaculatus* harbored a significantly higher abundance of amino acid metabolism and nucleotide metabolism genes (*P* < 0.05). The bacteria of *C. davidi* harbored a significantly higher abundance of carbohydrate metabolism genes (*P* < 0.05). The group of *C. bimaculatus* feeding on *Ca. tibetana* had a higher relative abundance than the groups of *C. davidi* feeding on *Ca. tibetana* and *C. bimaculatus* feeding on *Ca. sclerophylla* in the biosynthesis of other secondary metabolites pathway (*P* < 0.05).

There was no significant variation in the high abundance of genera involved in five metabolic pathways (Figure 5B). *Sodalis*, *Wolbachia*, *Klebsiella*, and *Rickettsia* in the phylum Proteobacteria were the main contributors to these pathways. The contribution of *Klebsiella* was reduced in nucleotide metabolism, carbohydrate metabolism, and biosynthesis of other secondary metabolites pathways. *Klebsiella* contributed to pathways of global and overview maps and amino acid metabolism of *C. bimaculatus* but had lesser contributions in *C. davidi*. In *C. davidi*, *Sodalis* with the highest abundance (Supplementary Figure 2) was the main contributor to these pathways. *Wolbachia* contributed low levels to the amino acid metabolism and carbohydrate metabolism pathways of *Ca. tibetana* feeders while being the leading contributor to nucleotide metabolism and biosynthesis of other secondary metabolites pathways. The contributions of *Wolbachia* in the two groups of *C. bimaculatus* were similar.

The metagenomic binning yielded four medium-quality non-redundant MAGs, three of which were associated with BGCs. No MAGs were identified in the *C. bimaculatus* feeding on *Ca. tibetana* (Figure 5C). The MAG unrelated to secondary metabolite biosynthetic gene clusters was classified as genus *Wolbachia* and was exclusively found in the *C. bimaculatus* feeding on *Ca. sclerophylla*. The three BGC-associated MAGs were classified as genus *Sodalis*. AntiSMASH analysis identified three types of BGCs within these MAGs: hserlactone, phosphonate, and T3PKS (Supplementary Table 5). Compared to the representative symbiotic bacterium *Sodalis pierantonius* of rice weevil *Sitophilus oryzae* (SOPE), the three *Sodalis* MAGs identified in this study lacked thiopeptide-BGCs, but contained an additional T3PKS-BGCs.

### Comparisons of metabolic enzymes, development, and detoxification of plant secondary compounds

**FIGURE 6:**
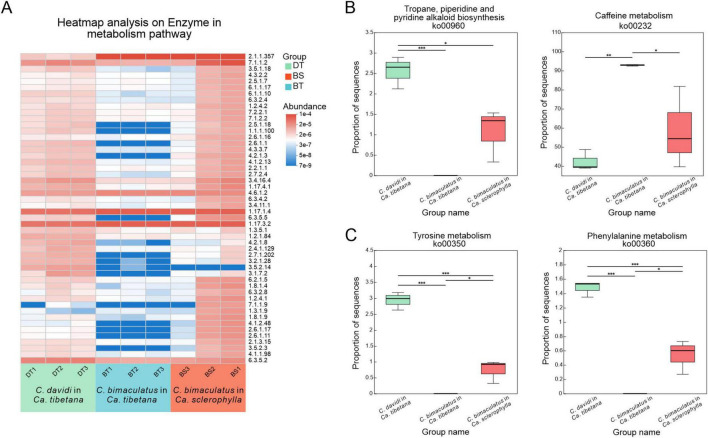
Comparisons of predicted KEGG ortholog group counts at enzyme levels and level 3 in metabolism pathway. **(A)** Heat map of the most abundant 50 enzymes in metabolism pathway. Each column corresponds to a *Curculio* sample, and each row corresponds to an enzyme. **(B)** Relative abundance of two key level 3 pathways involved in biosynthesis of other secondary metabolites pathway. **(C)** Relative abundance of two key level 3 pathways involved in amino acid metabolism pathway. (Kruskal–Wallis test; *P < 0.05, **P < 0.01, ***P < 0.001).

The patterns of variation in the abundances of 50 key enzymes were identified to understand the functions of enzyme responses in weevil metabolism. Almost all key enzymes were abundant in the groups of *C. davidi* feeding on *Ca. tibetana* and *C. bimaculatus* feeding on *Ca. sclerophylla*, while the group of *C. bimaculatus* feeding on *Ca. tibetana* exhibited low abundance of the metabolic enzymes (Figure 6A). Terpenoids, glucosinolates, and alkaloids are among the best-studied classes of plant secondary compounds. Thus, two key pathways involved in the biosynthesis of plant secondary compounds were also compared. The abundance of ko00960 (tropane, piperidine, and pyridine alkaloid biosynthesis) was significantly higher in *C. davidi* (Kruskal–Wallis H test, *P* < 0.05). In contrast, the abundance of ko00232 (Caffeine metabolism) in the group of *C. bimaculatus* feeding on *Ca. tibetana* was significantly higher than that in the groups of *C. davidi* feeding on *Ca. tibetana* and *C. bimaculatus* feeding on *Ca. sclerophylla* (Figure 6B). Tyrosine and phenylalanine are key aromatic amino acids involved in the exoskeleton development of beetles. The abundance of ko00350 (Tyrosine metabolism) and ko00360 (Phenylalanine metabolism) pathways was compared. *C. davidi* had a significantly higher abundance of these two key pathways. For *C. bimaculatus*, *Ca. sclerophylla* feeders had significantly higher abundances of the two pathways (Figure 6C).

## Discussion

Insect herbivores have significant effects on terrestrial ecosystems. However, specialized herbivory faces the problem of plant nutrients existing in a matrix of indigestible structural compounds such as cellulose, hemicellulose, and pectin as well as a variety of toxins and secondary metabolites (McNeil et al., 1984; Berenbaum, 1995; Wetzel et al., 2023). The seeds of plants in the Fagaceae are widely preyed on by phytophagous insects, birds, and rodents due to their richness in nutrients such as starch, soluble sugars, fats, and proteins (Dixon et al., 1997; He et al., 2022), while acorns also contain high levels of polyphenols, alkaloids, and flavonoids as secondary metabolites. The quantitative or qualitative changes in these phytochemicals influence predator selection preference and survival. However, microbes have bridged these metabolic gaps by promoting host insects’ tolerance to plant secondary metabolites such as terpenes, caffeine, nicotine, cocaine, isothiocyanates, and pesticides containing phosphorus or sulfur (Fan et al., 2011; Narasimhan et al., 2012; Marmulla and Harder, 2014; Wu et al., 2020). In this study, we determined the differences in the metabolite contents of *Ca. sclerophylla* and *Ca. tibetana*. Then, we annotated and compared the symbiotic bacteria in *C. bimaculatus* feeding on *Ca. sclerophylla*, *C. bimaculatus* feeding on *Ca. tibetana*, and *C. davidi* feeding on *Ca. tibetana* using 16S rRNA gene sequences. Functional predictions for the bacteria were then obtained by metabolomics analyses.

The bacterial phyla in weevil larvae were dominated by Proteobacteria, Firmicutes, and Actinobacteriota. Host plants had a strong influence on the diversity of symbiotic bacteria, whereas the host insects primarily influenced the community structure of the symbiotic bacteria. There were more similar species shared between the two groups of *C. bimaculatus*. Host plants play an important role in shaping the structure of symbiotic bacterial communities in phytophagous insects. Host tree species influenced the gut bacterial communities in *Monochamus saltuarius* (Coleoptera: Cerambycidae) larvae, but the Bray–Curtis distance of trees with similar metabolite abundance was low (Ge et al., 2021). The community structures of the bacterial genera differed significantly between strains of the pear lace bug *Stephanitis nashi* that fed on *Malus* spp. and *Cerasus* spp., while the metabolites of the two plants were very different. In this study, the abundances of most of the main bacterial genera were correlated with the metabolite contents. The prevalences of the genera *Sodalis*, *Candidatus* Curculioniphilus, *Methylorubrum*, *Rickettsia, Paenibacillus*, and *Sphingomonas* was 100%. *Candidatus* Curculioniphilus is the primary endosymbiont of *Curculio* spp., and it is an intracellular symbiont that is transmitted vertically via transovarial passage (Toju et al., 2010, 2013). Primary endosymbionts are closely related to their hosts, and they often illustrate the evolutionary trajectory of the host (Obata et al., 2011; Salem et al., 2017). The SOPE provides the tyrosine and phenylalanine essential for the cuticle development of the rice weevil *Sitophilus oryzae* (Coleoptera: Curculionidae), and it significantly enhanced the eclosion rate of the weevil (Anbutsu et al., 2017; Hirota et al., 2017). The functions of *Sodalis* in *Curculio* weevils may be similar. Meanwhile, the *Sodalis* MAGs assembled from the acorn weevil was found to contain a T3PKS (Type III Polyketide Synthase) BGC that is absent in SOPE (Supplementary Table 5). Compounds synthesized by T3PKS are typically small, structurally simple polyketides with cyclic structures, such as coumarins and flavonoids. This may be attributed to the higher abundance of plant secondary metabolites, such as flavonoids, in acorns compared to rice.

The COG and KEGG analyses of bacteria in *Curculio* weevils demonstrated their pivotal role in the metabolism of the hosts. The genes were enriched in amino acid metabolism and nucleotide metabolism pathways in *C. davidi* feeding on *Ca. tibetana*. Tyrosine and phenylalanine metabolism was exceptionally abundant. The indurative elytra and exoskeleton provide Coleoptera with enhanced mechanical protection, increase their resistance to desiccation and pathogenic infestations, and provide defense against predators (Linz et al., 2016; Goczał and Beutel, 2023; García-Lozano and Salem, 2024). Tyrosine and L-phenylalanine are precursor amino acids involved in the cuticle development of beetles; tyrosine must be taken in through the diet or from microbes (Noh et al., 2016; Anbutsu et al., 2017). In this study, *C. bimaculatus* feeding on *Ca. tibetana* had low enrichment of tyrosine metabolism and phenylalanine metabolism pathways, and this possibly led to the failure of eclosion. This may have been due to the extremely low abundance of *Sodalis* in the *C. bimaculatus* feeding on *Ca. sclerophylla*. *C. davidi* feeding on *Ca. tibetana* had significantly higher enrichment of tyrosine metabolism and phenylalanine metabolism pathways, with the highest enrichment of *Sodalis* bacteria. This connection suggests that the *Sodalis* in *Curculio* weevils performs the same function as in *Sitophilus* weevils (Anbutsu et al., 2017; Hirota et al., 2017).

As for the biosynthesis of other secondary metabolites, tropane, piperidine, and pyridine alkaloid biosynthesis pathway genes were less enriched in the group of *C. bimaculatus* feeding on *Ca. tibetana*, while caffeine metabolism genes were highly enriched in this group. Caffeine is an alkaloid that is toxic to many insect groups. The coffee berry borer (*Hypothenemus hampei*) harbored *Pseudomonas* that encoded and expressed caffeine demethylase genes to detoxify the caffeine (Ceja-Navarro et al., 2015). *Pseudomonas* contributes to the biosynthesis of other secondary metabolites in *Curculio* weevils. The contributions of bacteria to the caffeine metabolism of *Curculio* weevils remain unknown. The polyphagous *C. bimaculatus* can feed on most species of *Castanopsis*, *Lithocarpus*, *Quercus*, and *Castanea*, while the oligophagous *C. davidi* could only feed on several species of *Castanopsis* and *Castanea*. Overall, *C. bimaculatus* showed a wider range of adaptations in a number of different species. In this study, *C. bimaculatus* fed on *Ca. sclerophylla* and *Castanopsis tibetana*, while *C. davidi* only fed on *Ca. tibetana*. The *C. bimaculatus* strain feeding on *Ca. sclerophylla* was the dominant species in the population of *Curculio* weevils feeding on *Ca. sclerophylla*. However, *C. bimaculatus* was extremely underrepresented in the *Curculio* weevil population feeding on *Ca. tibetana*, and *C. davidi* was the dominant species among the *Ca. tibetana* feeders (unpublished data). The dominant genus of bacteria in *C. bimaculatus* feeding on *Ca. tibetana* was *Candidatus* Curculioniphilus, a microbial symbiont that is essential for development and reproduction of *Curculio* weevils, while other genera were present in lower proportions. This may result in the other genera not being able to perform their functions. For instance, the absence of *Sodalis* containing the T3PKS BGC in *C. bimaculatus* feeding on *Ca. tibetana* may indicate an inability to adapt to the flavonoid-rich environment of acorns (Figure 5C and Supplementary Table 5). Considering the absence of genes for tyrosine and phenylalanine metabolism and the enrichment of genes for caffeine metabolism (Figure 6), this strain of *C. bimaculatus* may have recently shifted to *Ca. tibetana* from a caffeine-containing plant, and it has not yet adapted to *Ca. tibetana*.

In summary, our results have demonstrated the differences in bacterial diversity and community structure among various host plant and host insect combinations. The host plants influenced the diversity of symbiotic bacteria, while the host insects influenced the community structure of symbiotic bacteria. The predictions made by metabolomics analyses further clarified the functions of bacteria in two dominant strains (*C. davidi* feeding on *Ca. tibetana* and *C. bimaculatus* feeding on *Ca. sclerophylla*), suggesting that bacteria have played a key role in host adaptation in *Curculio* weevils. *Sodalis*, *Wolbachia*, and *Klebsiella* were the main contributors to the metabolism. *Sodalis* plays an important role in the adaptation of plant secondary metabolites. The contribution of *Klebsiella* was centered on amino acid metabolism. *Wolbachia* was the leading contributor to nucleotide metabolism and biosynthesis of other secondary metabolites pathways of *Ca. tibetana* feeders. Further research is necessary explore the specific functions of major bacterial genera involved in host adaptation by removing the bacteria through antibiotic treatment.

## Data Availability

The datasets presented in this study can be found in online repositories. The names of the repository/repositories and accession number(s) can be found below: https://www.ncbi.nlm.nih.gov/, accession numbers PRJNA1194056 and PRJNA1194314.

## Footnotes

1
https://github.com/opengene/fastp

2
https://github.com/voutcn/megahit

3
https://github.com/hyattpd/prodigal

4
http://www.bioinformatics.org/cd-hit/

5
https://github.com/ShujiaHuang/SOAPaligner

6
https://github.com/bbuchfink/diamond

7
https://github.com/binpro/concoct

8
https://github.com/benlangmead/bowtie2/releases/tag/v2.2.9

9
https://github.com/ecogenomics/checkm

## Supplementary material

Supplementary Figure 1 **(A)** Comparison of Alpha diversity of bacterial communities (Kruskal–Wallis test; **P* < 0.05, ***P* < 0.01, ****P* < 0.001). **(B)** Network analysis applied to the bacterial genera of *Curculio* spp. Node sizes represent relative abundance of the genera. The top 11 dominating genera were labeled. **(C)** Hierarchical clustering tree on genus level of different samples (based on Bray–Curtis distance).

Supplementary Figure 2 Community analysis pieplot on genus level of three groups.

Supplementary Figure 3 **(A)** Microbiological composition of different samples. **(B)** Hierarchical clustering of bacterial samples based on COG and KEGG data (based on Bray–Curtis distance).

Supplementary Table 1 The alpha diversity index of symbiotic bacteria of weevils.

Supplementary Table 2 ASVs annotation of symbiotic bacteria of weevils.

Supplementary Table 3 COG annotation of symbiotic bacteria of weevils.

Supplementary Table 4 KEGG annotation of symbiotic bacteria of weevils.

Supplementary Table 5 Result of metagenomic binning and antiSMASH analysis.
