# Supplementary figures and images for: Symbiotic bacteria associated with different species of Curculio (Coleoptera: Curculionidae) and their host plants
Source: Front Microbiol. 2025 Mar 14;16:1531847. doi: 10.3389/fmicb.2025.1531847 (PMC11952766; doi:10.3389/fmicb.2025.1531847)

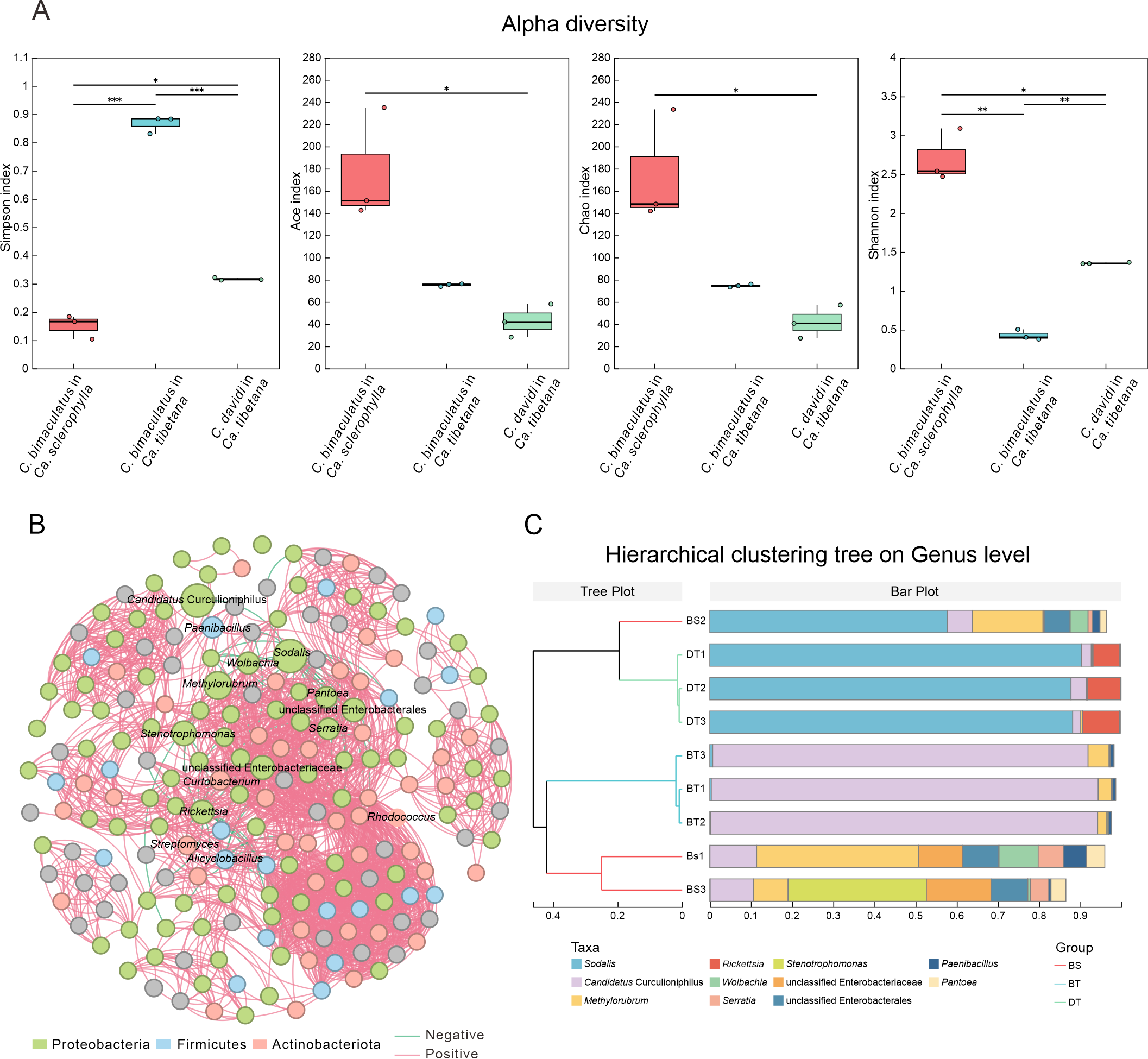

Supplement: Supplementary Figure 1 — (A) Comparison of Alpha diversity of bacterial communities (Kruskal–Wallis test; *P < 0.05, **P < 0.01, ***P < 0.001). (B) Network analysis applied to the bacterial genera of Curculio spp. Node sizes represent relative abundance of the genera. The top 11 dominating genera were labeled. (C) Hierarchical clustering tree on genus level of different samples (based on Bray–Curtis distance). [file Image_1.tif]

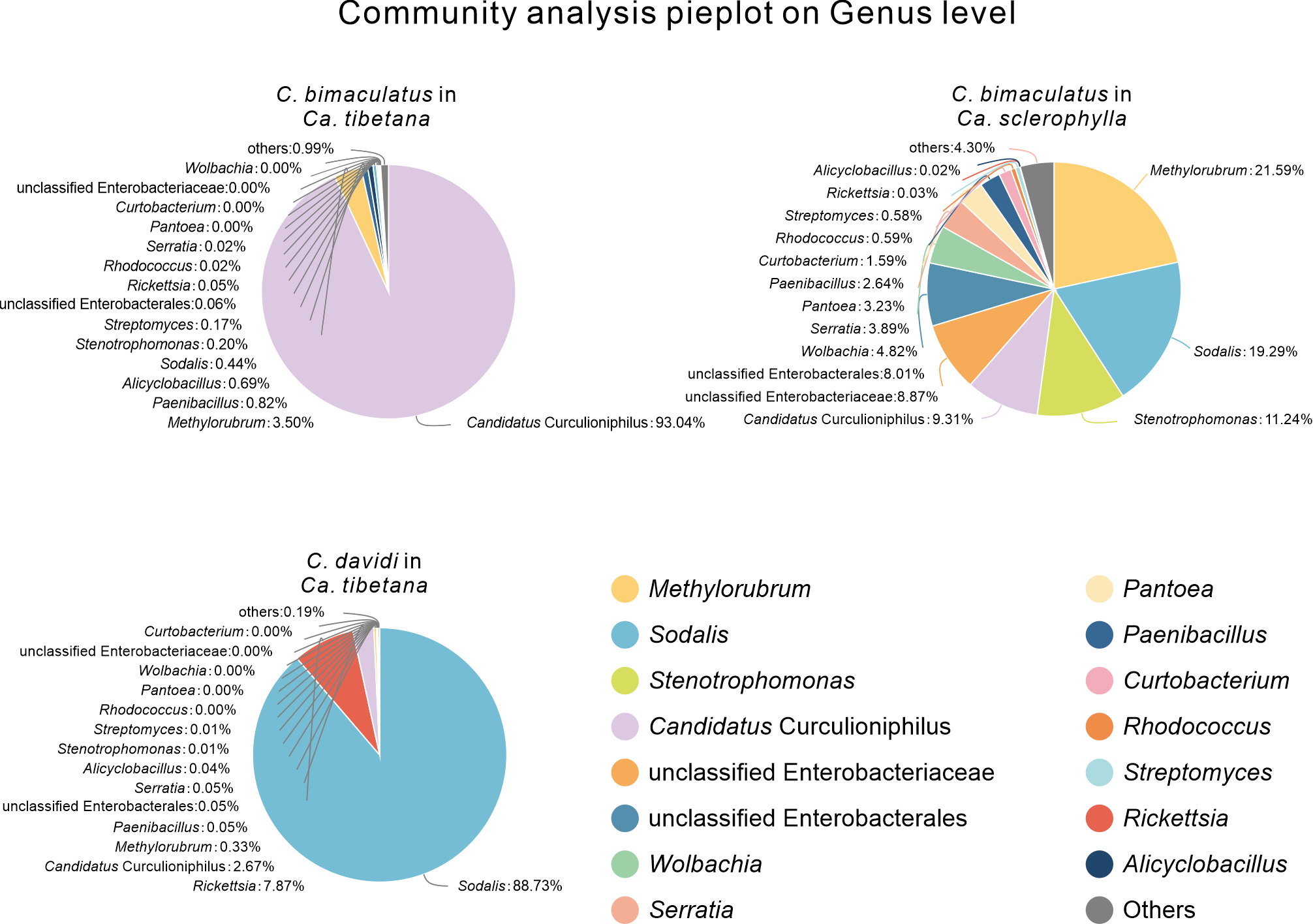

Supplement: Supplementary Figure 2 — Community analysis pieplot on genus level of three groups. [file Image_2.tif]

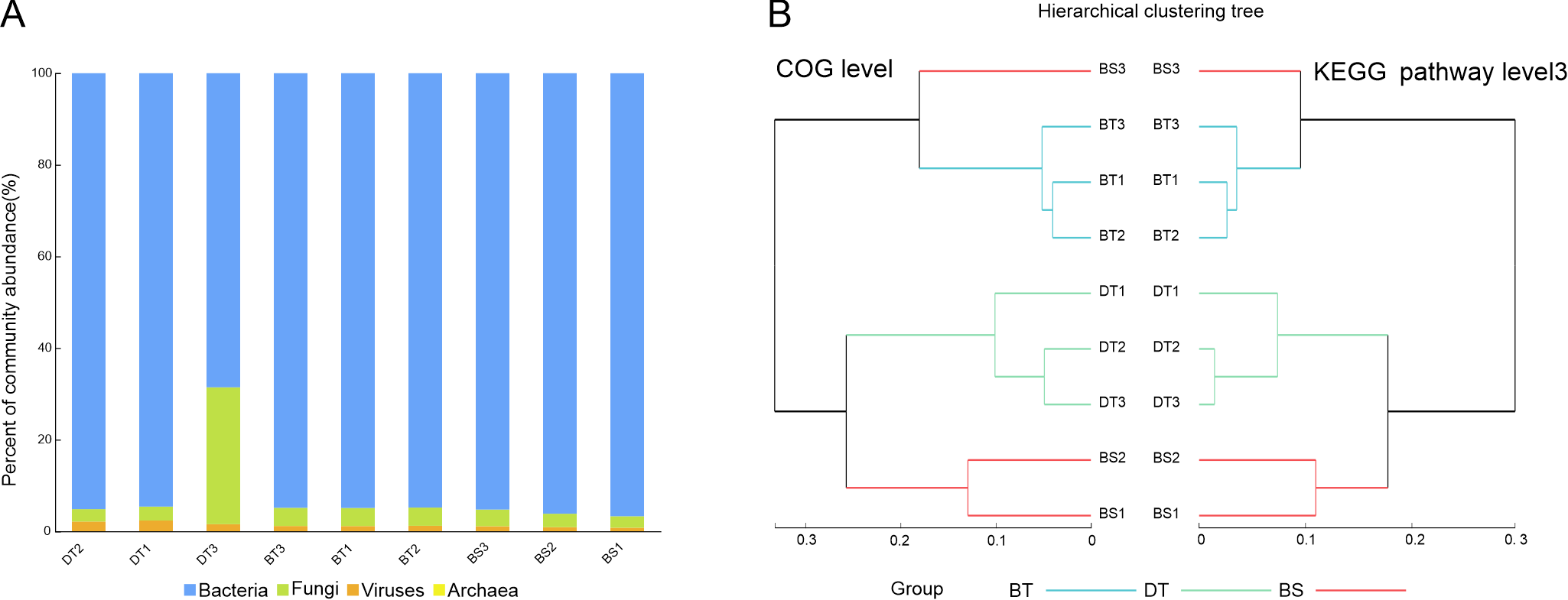

Supplement: Supplementary Figure 3 — (A) Microbiological composition of different samples. (B) Hierarchical clustering of bacterial samples based on COG and KEGG data (based on Bray–Curtis distance). [file Image_3.tif]
